# Long-term survival of an adolescent glioblastoma patient under treatment with vinblastine and valproic acid illustrates importance of methylation profiling

**DOI:** 10.1007/s00381-021-05278-6

**Published:** 2021-07-26

**Authors:** Catena Kresbach, Annika Bronsema, Helena Guerreiro, Stefan Rutkowski, Ulrich Schüller, Beate Winkler

**Affiliations:** 1 Department of Pediatric Hematology and Oncology, University Medical Center Hamburg-Eppendorf, Martinistrasse 52, O 47, 20251 Hamburg, Germany; 2 Research Institute Children’s Cancer Center Hamburg, Hamburg, Germany; 3 Department of Diagnostic and Interventional Neuroradiology, University Medical Center Hamburg-Eppendorf, Martinistrasse 52, 20246 Hamburg, Germany; 4 Institute of Neuropathology, University Medical Center Hamburg-Eppendorf, Hamburg, Germany

**Keywords:** Glioblastoma, Vinblastine, Valproic acid, Methylation profiling

## Abstract

Glioblastoma (GBM) is an exceptionally aggressive brain tumor with a dismal prognosis, demanding fast and precise classification as a base for patient-specific treatment strategies. Here, we report on an adolescent patient with a histologically *bona fide* GBM that shows a molecular methylation profile suggesting a low-grade glioma-like subgroup. Despite an early relapse, intolerance of temozolomide, and change of treatment strategy to vinblastine and valproic acid (VPA), the patient is now in good clinical condition after more than 5 years since initial diagnosis. This case stresses the merit of methylation array data for clinical prognosis and treatment planning.

## Introduction

High-grade glioma accounts for 10–15% of pediatric CNS tumors [1]. Gross surgical resection and radiotherapy are essential for survival. A rational base for chemotherapy treatment, however, is still missing [2]. The current German HIT HGG 2013 study investigates an additive effect of valproic acid (VPA) to temozolomide and radiotherapy [3]. But despite multimodal therapy approaches, the prognosis of pediatric glioblastoma patients with high-grade GBM is extremely poor. As prognosis varies significantly in dependence of clinical and biological features, identification of reliable prognostic parameters for treatment response and clinical outcome of individual patients is urgent [4].

DNA methylation profiling contributes to define molecular subgroups in pediatric GBM that are clinically and prognostically important [5, 6]. Two independent investigations in 2015 and 2017 describe that a subset of H3/IDH wild-type GBM showed methylation profiles resembling low-grade glioma (LGG-like) or pleomorphic xanthoastrocytoma (PXA-like). The LGG-like tumors occurred mainly in infants under the age of 1 year and showed a significantly better prognosis than other H3/IDH wild-type GBM [5, 6].

## Case report

**Fig. 1:**
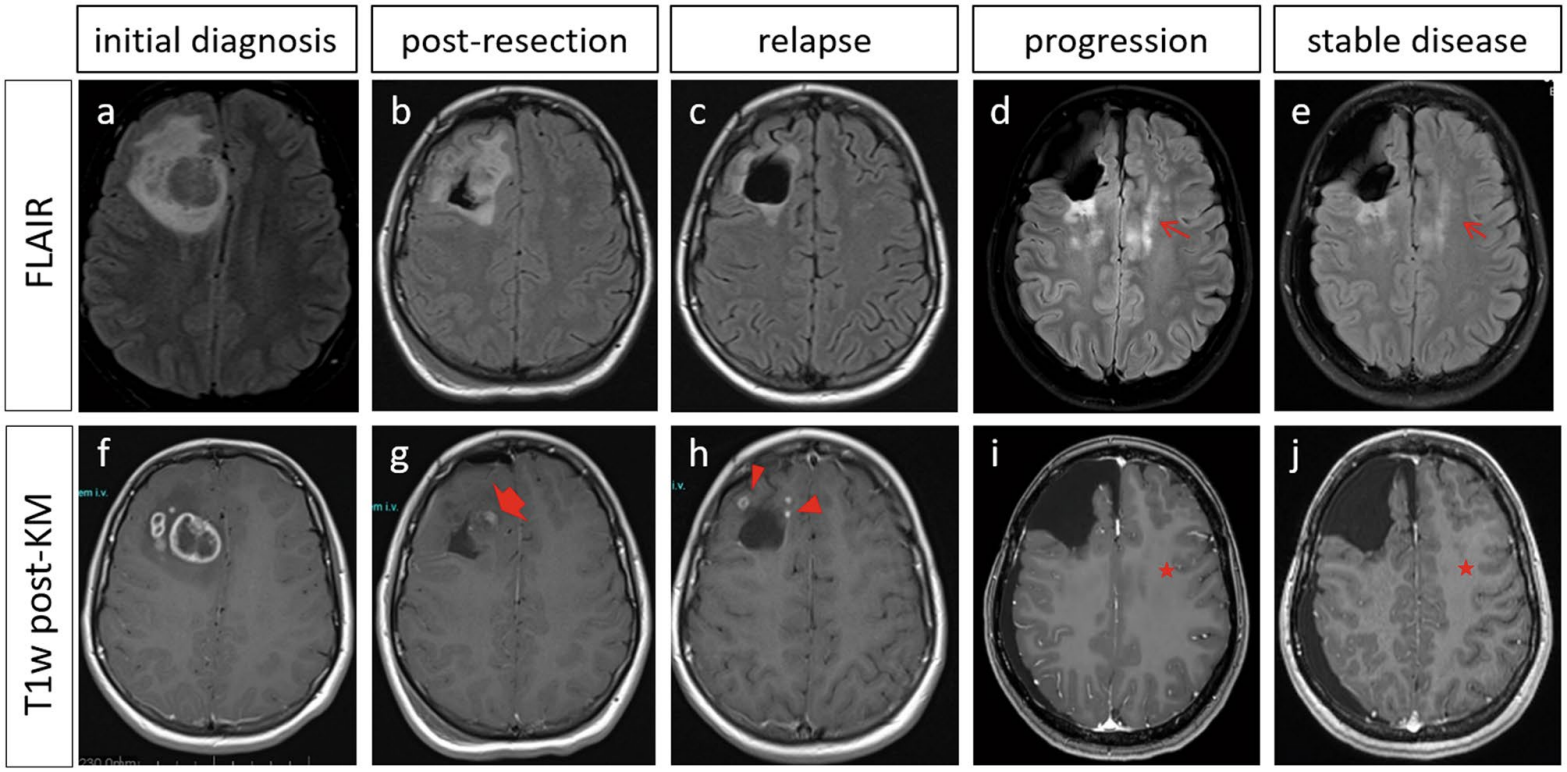
Cranial MRIs before and during treatment: T2 fluid-attenuated inversion recovery (FLAIR) **a**–**e** and contrast-enhanced T1 weighted **f**–**j** MRI sequences. Upon initial diagnosis **a**, **f**: heterogeneous mass on the right frontal lobe with perifocal edema **a**, ring enhancement **f**, a slight midline deviation and the presence of satellite lesions **f**. Post-operative imaging **b**, **g**: 2 days after tumor resection showing typical perifocal edema **b** and a focal nodular enhancement **g** at the anteromedial resection margin (broad arrow) suggesting possible residual tumor. Upon relapse 7 months after tumor resection **c**, **h**: frontal defect with discrete perifocal edema **c** and relapsing contrast-enhancing lesions (triangles) adjacent to the resection area **h**. Upon progression **d**, **i**: larger post-resection parenchyma defect on the right frontal lobe after repeated tumor resection and progressive hyperintense signal in the FLAIR **d** in the contralateral cingulate gyrus (arrow). Note the absence of enhancement (star) in the post-contrast images **i**. Seven months after progression, MRI images show stable disease **e**, **j**: stable non-enhancing (star) FLAIR hyperintensities (arrow) in the left cingulate gyrus **e**

The 15-year-old female patient presented at the Department of Pediatric Hematology and Oncology, University Medical Center Hamburg-Eppendorf with several weeks of frontotemporal headache and was admitted to the hospital with transient weakness and paresthesia of the left extremities. The cranial MRI showed a right-frontal tumor with surrounding edema (Fig. 1a, f).

**Fig. 2:**
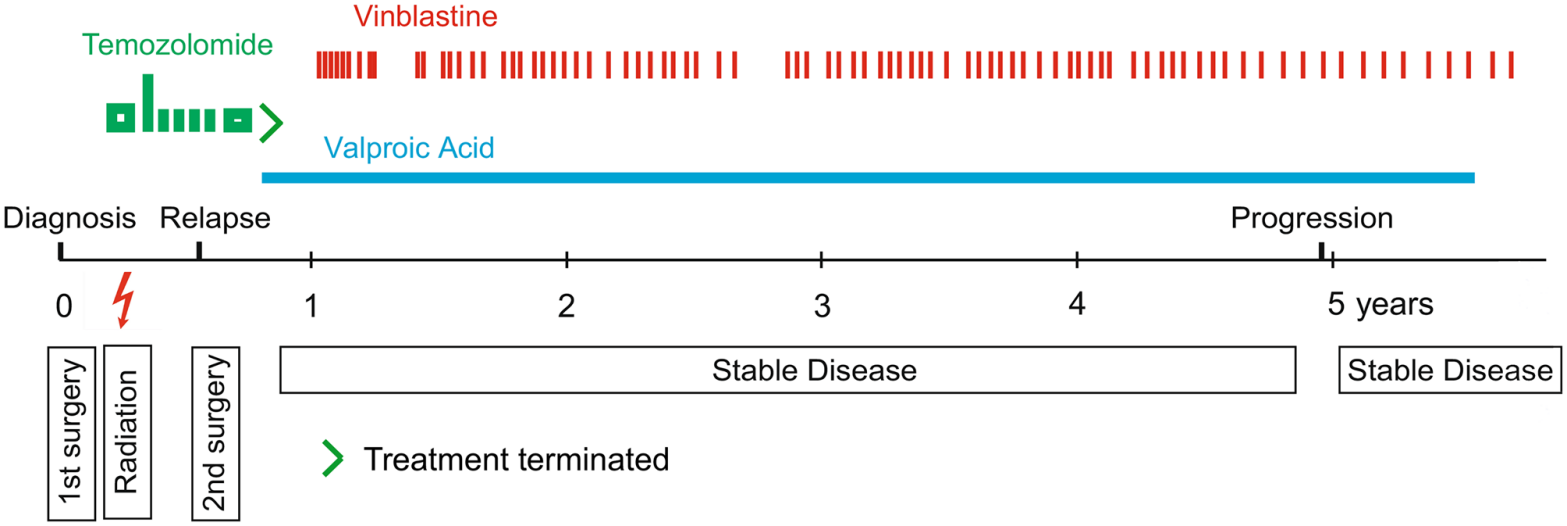
Time line showing patient’s treatment. Chemotherapeutical treatment: oral temozolomide (green) was given concomitant to radiotherapy and then as a monotherapy first 150 mg/m^2^/day (5 days every 4 weeks), then 100 mg/m^2^/day and was terminated due to severe side effects. Valproic acid (blue) 300 mg/m^2^/day in 2 daily oral doses and vinblastine 3 mg/m^2^ treatment (red) was added first as weekly infusions and then in longer intervals of 2–3 weeks. Dosages remained unchanged for the entire treatment period

After macroscopically complete tumor resection, a small residual tumor was suspected in the postoperative MRI (Fig. 1b, g). Histomorphologic assessment by local and reference pathologists confirmed the diagnosis of IDH-mutant GBM. The tumor region was irradiated with a target volume dose of 60 Gy following the HIT HGG-2007 protocol with concomitant daily temozolomide, followed by a temozolomide monotherapy in monthly cycles of 5 days (treatment overview in Fig. 2). Seven months after diagnosis, the tumor reoccurred (Fig. 1c, h). After the second surgical resection, a residual tumor at the dorsal resection border could not be excluded. We initiated adjuvant therapy with oral valproic acid (VPA) and due to slight progression of the residual mass, we added treatment with weekly vinblastine. At the time of 4.5 years after the first diagnosis, Vinblastine and VPA dosage remained unchanged but vinblastine applications were stretched to 3-week intervals in order to reduce adverse reactions.

Five years after initial diagnosis and 6 months after decreasing the treatment intensity, MRI imaging showed a progressive hyperintense signal in the FLAIR sequence in the contralateral cingulate gyrus, indicating a new non-enhancing tumor lesion (Fig. 1d, i). The patient was clinically well and very hesitant towards intensified chemotherapy or surgery. Therefore, and with respect to the slow progression, we decided to continue the previous treatment schedule with VPA and vinblastine and to closely surveille the tumor by MRI imaging. The tumor showed no further progression on MRI for the following 7 months (Fig. 1 e, j).

## Neuropathological and molecular workup

**Fig. 3:**
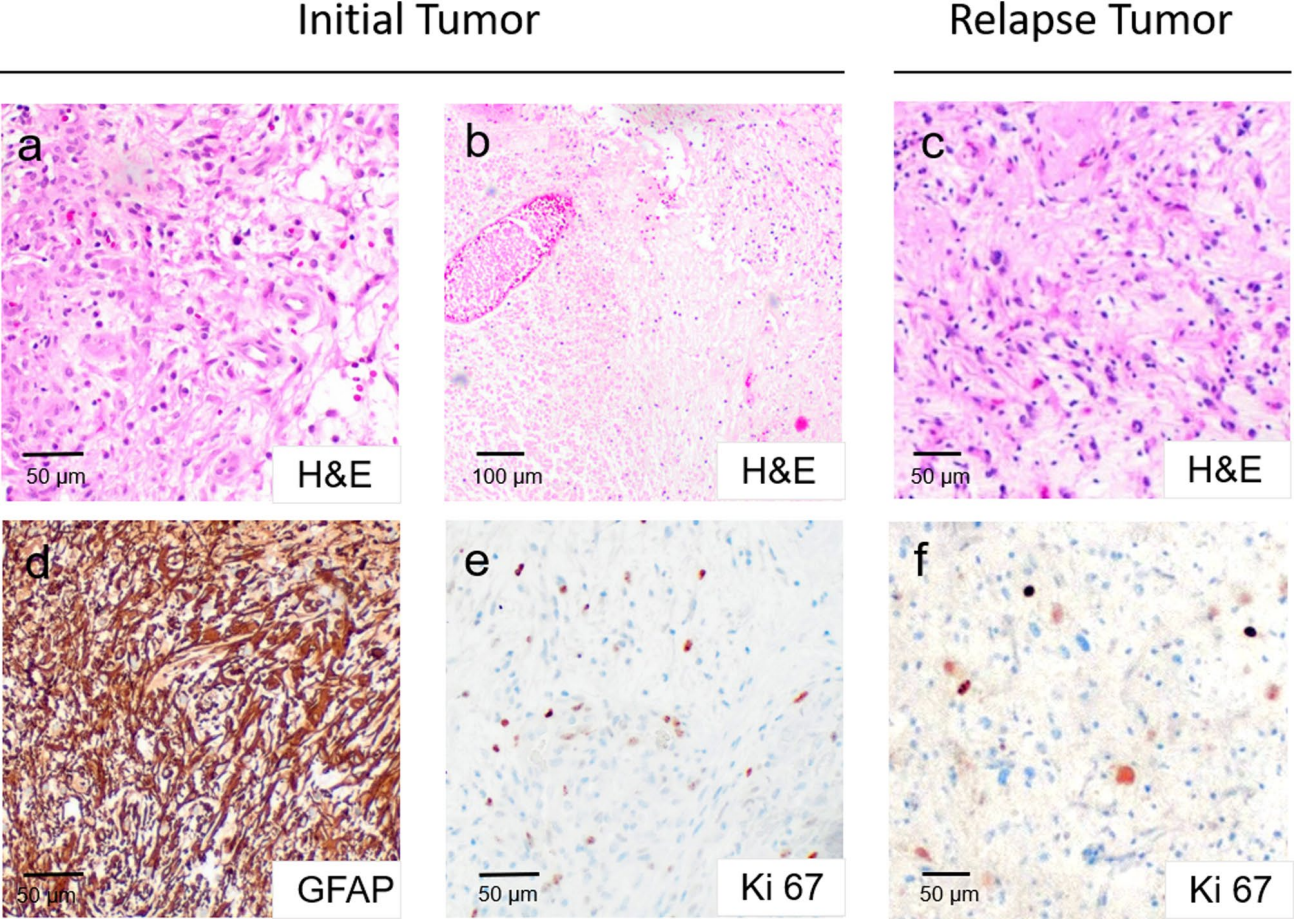
Histology of initial tumor (left) and relapse tumor (right): glial tumor with vascular proliferation and necrosis **a**–**c**. Positive staining of GFAP **d**. 10–20% Ki67-positive cells in initial and recurrent tumor **e**, **f**. Histological diagnosis: Glioblastoma (°IV)

**Fig. 4:**
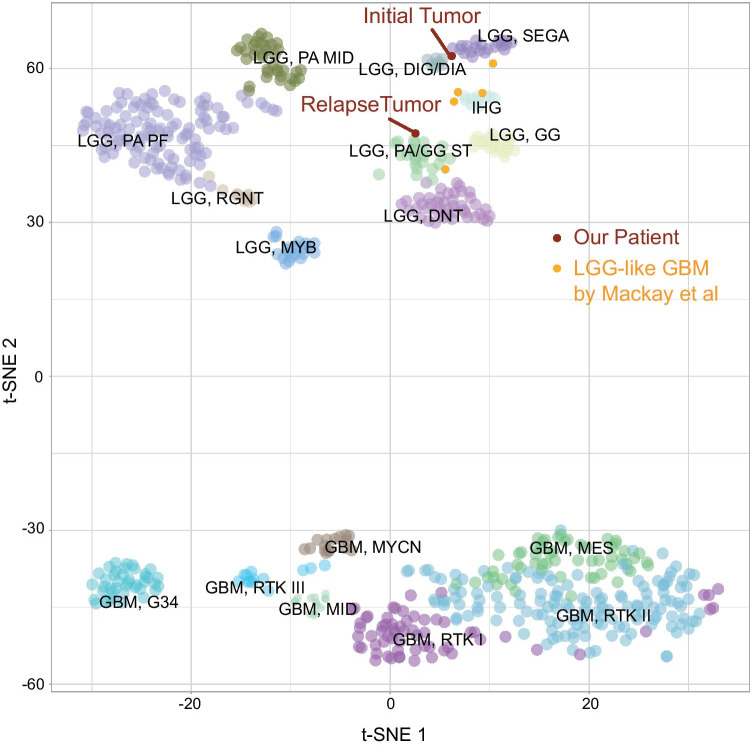
t-SNE analysis of the methylation profile suggested similarity to low-grade glioma: t-SNE plot including glioblastoma and low-grade glioma from the Heidelberg brain tumor classifier as published by Capper et al. [7]. Primary and relapse tumor of our patient depicted in red. Exemplary LGG-like glioblastoma from Mackay et al. depicted in orange [6]

We investigated the biological characteristics of the tumors in more detail (Figs. 3 and 4). The initial histology showed a glial tumor with vascular proliferation, areas of necrosis, and increased proliferation. Tumor cells were positive for GFAP and MAP2c and negative for IDH-1 (R132H) and mutation-specific Histone H3. DNA sequencing ruled out mutations in codon 27 or 34 of the *H3F3A* gene.

We conducted Illumina Human Methylation 850 K Bead Chip array analysis of primary and relapse tumor tissue. Both tumors showed an unmethylated MGMT promoter. The tumor did not show a definite match with any of the reference brain tumor entities on the Heidelberg brain tumor classifier [7]. The initial tumor had a calibrated score of 0.4, and the relapse tumor did not score above 0.3. We then performed t-distributed stochastic neighbor embedding (*t*-SNE) analysis alongside 2,801 brain tumor cases as published [7]. Initial and relapse tumor clustered closely with low-grade glioma, comparable to published methylation data of LGG-like GBM by Mackay et al. [6] (Fig. 4).

## Discussion

Despite an early relapse and a residual tumor, the adolescent patient remained with stable disease for over 5 years under treatment with vinblastine and VPA.

Weekly vinblastine has previously proven successful in treatment of recurrent LGG [8]. Vinca alkaloids showed activity in early chemotherapy studies [9, 10] and are a key element in previous and current treatment strategies [11].

VPA is a well-established anti-epileptic drug but was also found to induce an anti-tumor response in vitro and in GBM patients and has been proposed to have sensitizing effects during radiation therapy [12–15]. Due to promising preclinical and clinical reports, treatment with VPA is currently tested in the running German HIT-HGG 2013 trial [3].

Methylation analysis did not detect a clear match with any brain tumor methylation class, but *t*-SNE analysis of the methylation profile suggested similarity to low-grade glioma. The correct diagnosis of glioblastoma is strongly supported by the aggressive tumor biology and the clear histological features. However, the methylation profile in combination with the good response to VPA and Vinblastine treatment point towards this glioblastoma having some LGG-like features as described previously [5, 6].

Whether the good treatment response of our patient is due to an intrinsically less aggressive tumor biology, as suggested by the LGG-like methylation profile, or if the applied treatment strategy also contributed to the long-term survival remains unknown and will have to be put into context with detailed clinical data from comparable patients.

With respect to the devastating prognosis of GBM, we stress the importance of methylation profiling for risk stratification and treatment. Adolescent patients with GBM clustering in the LGG-like subgroup and an above-average prognosis might be more common than suggested by previous reports. Clustering of tumors in the LGG-like subgroup might even supply evidence for alternative treatment strategies including vinblastine and VPA.
